# Annotations

**Published:** 1903-03-28

**Authors:** 


### Annotations.

### The Eighteenth-Century Practitioner of Medicine.

Medicine rightly claims to be the most progressive of the applied sciences. It is an art which, although burdened by tradition and clouded by ignorance, still strives incessantly after a more perfect way. The wisdom and folly of the human is well exemplified by a study of the history of medicine. In the dawn of the twentieth century when, as we fondly hope and sometimes openly claim, our methods of healing are rational and dictated by the scientific spirit, it is well to turn to such interesting records of comparatively near times as are available in such a work as the late Sir Walter Besant's "London in the Eighteenth Century." In this charming study of a bygone age there is much of particular interest to the physician and student of medicine of to-day. In these days a man's profession could be diagnosed by his dress and demeanour. The physician in those, as they now seem, mediæval days thought it well to keep aloof from the common herd, seldom frequented coffee houses and maintained by seclusion something of the mystery which had formerly associated medicine with sorcery or at least astrology. But it is well to remember that oftentimes the physician was a scholar, and many gained distinction as collectors, antiquaries, numismatists, lovers of painting and statuary, or students of music, rather than by their success as healers of disease.

### More Schemes.

The midwifery movement promises, or threatens, to go further than even its warmest advocates anticipated. The latest proposal is to create a "general hospital with a 70 bed maternity annexe" in one of "those densely populated outlying London districts where such institutions are badly needed." In it is to be located a "national training school for midwives," whose students are to be "some of that large proportion of educated gentlewomen among us, who are anxious, while incidentally earning their own living, to do something worth doing." The training therein is to be 18 months of general nursing and six months of district maternity work, while after every three years of independent work, the "graduate" is to return for further examination and coaching in the advances of the obstetric art. The product of course is to be the ideal midwife, who is to solve all the difficulties of the existing situation as well as (incidentally) earn her own living. The letter in the *Times*, in which this idea is given birth, contains much evidence that its writers are enthusiastic, but little that they are in touch with leaders, either of medicine, nursing education, or midwifery reform. Without wishing to throw unnecessary cold water upon it, it may be pointed out that the Midwives' Board has not yet formulated its views of the training that future midwives should undergo, and that it will be quite time enough to talk of a new call upon the charitable resources of London when it is seen that existing lying-in hospitals and midwifery training schools are not inclined to give those views due effect as soon as they are known. The two definite factors that give life to the midwifery movement are the existence of women midwives who do not possess the skill with which they are credited by their patients, and the absence of any provision for the after-care of women of the poorer classes who have recently been confined. The distinction thus indicated between midwifery and midwifery nursing is one that is rarely sufficiently appreciated by reformers. The evil is a double one, and both parts of it will have to be met. The solution seems more likely to be found in an extension of district and village nursing system than by creation of an expensive hybrid with neither the responsible skill of a doctor nor the thorough training of an ordinary nurse. Any midwife would certainly be the better for being also a thoroughly trained nurse; but no successful midwife is likely to have time to thoroughly nurse her patients as well as efficiently deliver them, nor will she give up her work every third year to return to a school. After all, why should she?

### The Revolt in the Kindergarten.

There is little doubt that Frœbel, if he had lived in Tennyson's time, would have shared that poet's fear "lest one good custom should corrupt the world." Frœbel was a reformer, if not a revolutionary, and would have been the last to think that he had said the last word on education. Yet, since Frœbelian methods have come into fashion, there has been the natural tendency among his admirers to think that rules which he laid down as suggestions for teaching must be maintained with the inflexibility of Medo-Persic laws. He suggested paper-folding as an exercise for children's minds and hands, and paper folding has been continued through the generations with no change in idea, and modified only in the direction of growing more and more elaborate. Against this hygienists are now beginning to protest, and Miss E. M. Murray recently flung the apple of discord into the midst of the Frœbel Society itself by an essay which maintained that "Symmetrical Paper-folding and Symmetrical Work with the Gifts is a Waste of Time." Miss Murray's contention was that though this paper-folding did undoubtedly give some training in accuracy, equally good training could be given by other tasks which would, in the end, produce something which would be of permanent use or pleasure to the producer. A medical woman, Dr. Marion Gilchrist, tested the eyes ef the children in a large number of Glasgow schools, and found that only 55 6 per cent. of the children had full vision, and of these only 21·4 per cent. were really perfect. Of the 44·4 per cent. whose vision was defective nearly a half had "less than a half of full vision." Moreover to set those whose sight is defective to such work as the careful paper-folding of the kindergarten is cruelty, and cruelty of the most useless description, for instead of making the children accurate, the strain of the task will only intensify that weakness of sight which makes accuracy impossible, and may seriously injure the child for all his life. It would, therefore, be only reasonable that a child's eyes should be tested before he is set to work of this kind. And even if it is proved that the eyes can stand it, it is worth while to ask if the pupil's energies could not be turned to work which is at least equally educative and more obviously useful. Dr. Johnson described the fancy-work which ladies used to do as being "red with the blood of murdered time." The schooltime of children is too precious to be massacred in similar fashion.

